# *In vitro* and *in vivo* evaluation of electrophoresis-aided casein phosphopeptide-amorphous calcium phosphate remineralisation system on pH-cycling and acid-etching demineralised enamel

**DOI:** 10.1038/s41598-018-27304-5

**Published:** 2018-06-11

**Authors:** Yu Yuan Zhang, Hai Ming Wong, Colman P. J. McGrath, Quan Li Li

**Affiliations:** 1 Faculty of Dentistry, The University of Hong Kong, 34 Hospital Road, The Prince Philip Dental Hospital, Hong Kong, China; 2 Department of Prosthodontic, Collage and Hospital of stomatology, Anhui Medical University, Key Lab. of Oral Diseases Research of Anhui Province, Hefei, China

## Abstract

Casein phosphate-amorphous calcium phosphate (CPP-ACP), as a remineralisation agent, is extensively used in managing demineralised enamel; however, its remineralisation kinetics is low. This study aimed to improve remineralisation kinetics of CPP-ACP by introducing a rapid remineralisation method with electrophoresis. *In vitro*, a pH-cycling enamel model was used to test remineralisation potentials of electrophoresis-aided CPP-ACP. For verifying remineralisation potentials of electrophoresis-aided CPP-ACP *in vivo* in a rabbit model, acid-etched enamel surface on rabbit maxillary incisors was remineralised by electrophoresis-aided CPP-ACP with 1.0 mA (group A) or 0.5 mA (group B). Both *in vitro* and *in vivo*, it was observed that electrophoresis was benefit to improve remineralisation kinetics of CPP-ACP, and the demineralised enamel was completely remineralised after 5 h. The Ca/P ratio in remineralised enamel consisted with that of hydroxyapatite, the microstructure in native enamel. Meanwhile, *in vivo* the micro-hardness of acid-etched enamel in group A (322.55 ± 31.90) and group B (322.55 ± 31.90) recovered up to the value of native enamel after 5 h remineralisation (p > 0.05). The Hematoxylin-eosin stain demonstrated that the electric field used in this study was safe on rabbit dental pulp. Therefore, this efficient and safe method has the potential to be applied in treating enamel deminerlisation.

## Introduction

The tooth is the most highly mineralised and hardest tissue in the human body. It is composed of enamel and dentin-pulp complex. As the outmost layer of teeth, enamel contains hydroxyapatite in the form of well-organised prism pattern that can load the stresses associated with mastication and prevent dentin-pulp complex away from stimuli^1,2^.

Tooth demineralisation is a common problem worldwide due to the increase consumption of sugary snacks. The complex long-term interaction between acid-producing bacteria and fermentable carbohydrates results in surface loss of teeth and the development of dental caries. If caries is deep and the dentinal tubules are exposed, dentine hypersensitivity and even pulpal inflammation may occur^3^. Traditional treatment for dental caries is to remove decayed tissue and fill the cavity with dental restorative materials. Unfortunately, secondary caries caused by marginal leakage is frequently observed after a period of time. Thus, numerous non-invasive approaches, such as the local application of fluoride or remineralisation agents, have been investigated to promote remineralisation and prevent demineralisation for managing early stage of caries.

Casein phosphopeptide-amorphous calcium phosphate (CPP-ACP) is one of the commercially available remineralisation agents and is most commonly used^4,5^. Casein phosphopeptides (CPP) is tryptic multiphsphorylated peptides of caseins derived from milk and milk products, which can sustain high concentration of calcium and phosphate ions in metastable solution by forming CPP-ACP nanocomplexes. As a calcium-phosphate reservoir, CPP-ACP can depress enamel demineralisation and promote remineralisation^6^. Numerous studies have successfully proved the ability of CPP-ACP regarding prevention of demineralisation and promotion of remineralisation^7–10^. The ability of remineralisation for CPP-ACP was demonstrated to be superior than fluoride particularly in managing post-orthodontic white-spot lesions^11,12^. However, remineralising by CPP-ACP is time-consuming. It usually takes several days to achieve an appropriate coating. Therefore, more and more controversies with respect to the remineralisation potential of CPP-ACP have been raised^13–16^. For instance, the remineralisation potential of CPP-ACP was reported to be weaker compared with that of fluoride-based toothpastes^16^. The repair effect of CPP-ACP on the acid-etched enamel could also be limited, particularly on nanomechanical and anti-wear properties^17^. Most importantly, the remineralisation kinetics of CPP-ACP was shown to be very low^18^.

Electric field is an easy method to manipulate bioparticles with the advantages of strong controllability and high efficiency. Electrophoresis, including iontophoresis, can directly transport ions in teeth through untilising the direct electric field with a low amperage of direct electrical current^19^. Fluoride iontophoresis is a device that uses an electric-field pulse to deliver fluoride ions to the teeth. Plenty evidences have shown its effectiveness on promoting the effect of topical fluoride application for preventing dental caries and reducing dental hypersensitivity^20–22^.

Electrophoresis was introduced in this study to incorporate with CPP-ACP for accelerating the speed of remineralisation. Meanwhile, due to the limit evidence demonstrating the impact of electric field on dental pulp, an acid-etched rabbit incisor model was used to examine pulp histomorphology after the stimulation by electric field.

## Results

### The morphology and element compositions of new crystals formed on remineralised enamel

**Figure 1:**
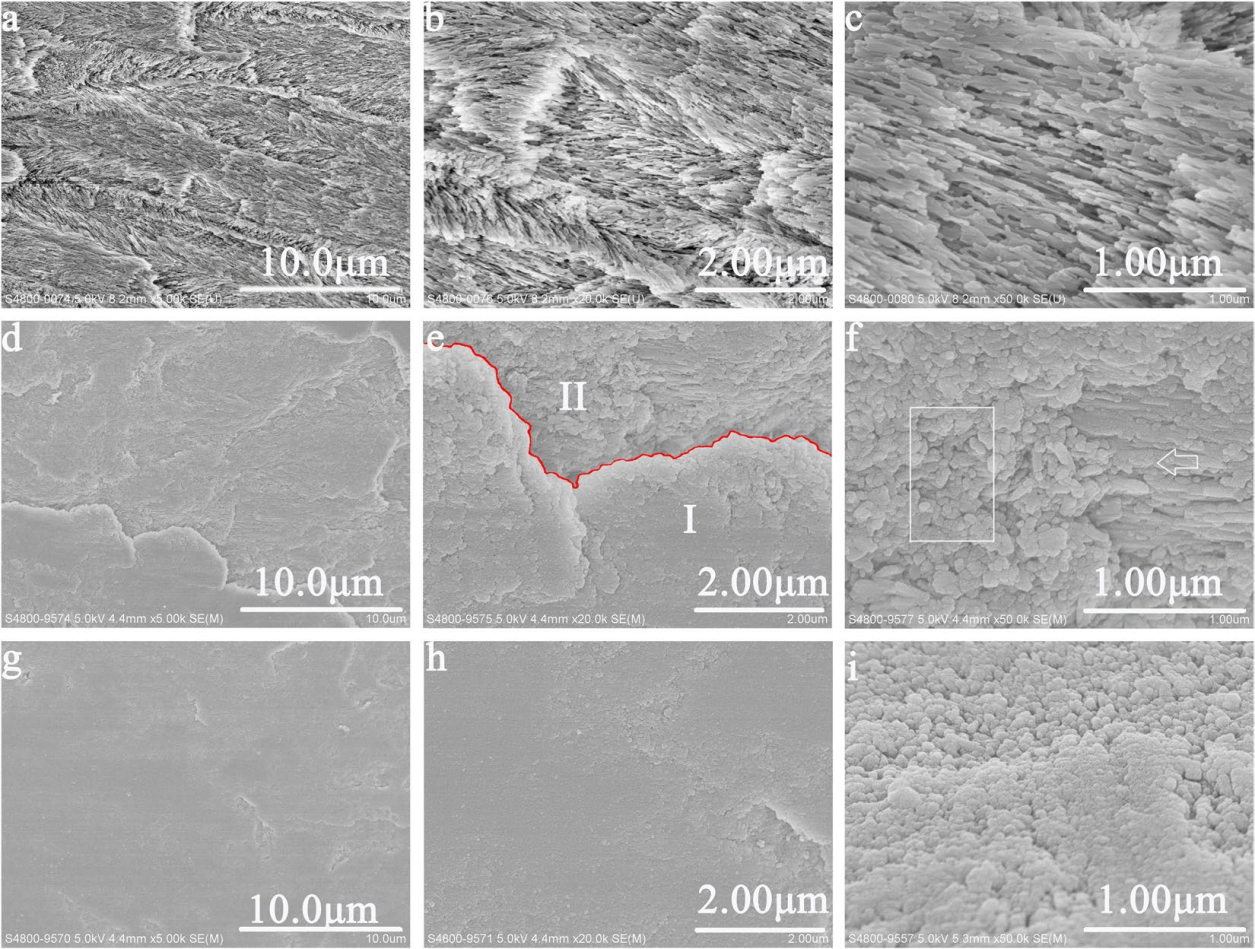
SEM micrographs of remineralised enamel *in vitro* study. (**a**) Remineralised enamel after 5 h remineralisation in control group; (**b**) and (**c**) The magnified micrograph of (a). (**d**) Remineralsied enamel after 3 h remineralisation in experimental group. (**e**) The magnified micrograph of (**d**) (Red line: boundary between new crystal layer (area I) and original enamel (area II)). (**f**) The magnified micrograph of area II (Rectangle: formed new crystals; arrow: self-growth of demineralised enamel). (**g**) Remineralsied enamel after 5 h remineralisation in experimental group (**h**) and (**i**)The magnified micrograph of (g).

*In vitro* study, Fig. 1 illustrated the remineralised enamel surfaces in experimental group and control group. According to the SEM observation, after 5 h remineralisation, many fragmentary crystals still existed on demineralised enamel in control group without the aid of electrophoresis. No formation of new crystals was observed (Fig. 1a–c). Conversely, in experimental group with the aid of electrophoresis, numerous crystals formed on demineralised enamel at the end of 3 h remineralisation (Fig. 1d–f). With the increased remineralisation time, more and more crystals have formed on the surface of demineralised enamel (Fig. 1; Supplementary file). At the end of 5 h remineralisation, the surface of demineralised enamel was fully remineralised and covered by a thick layer of new crystals (Fig. 1g–i).

**Figure 2:**
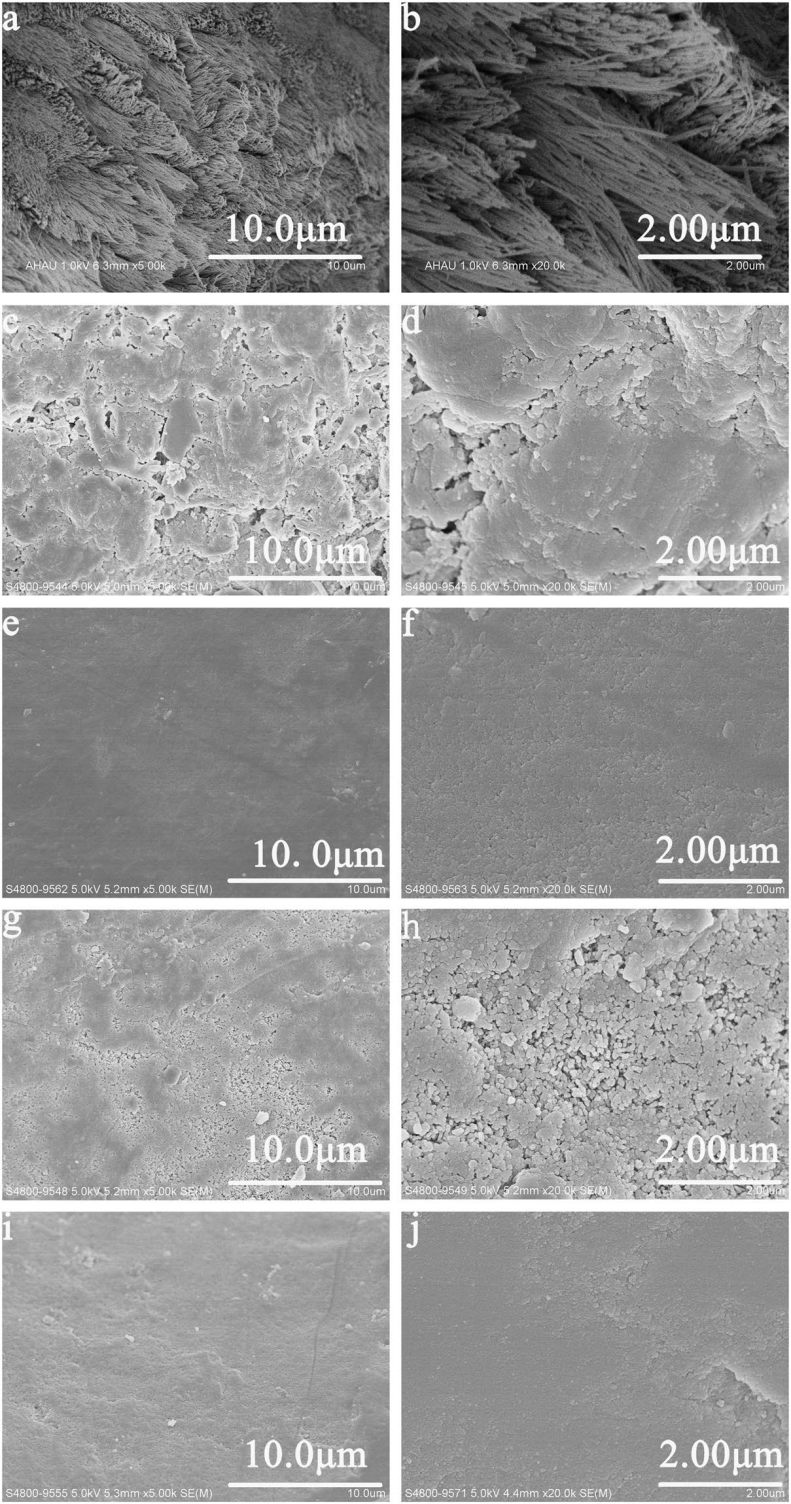
SEM micrographs of acid-etched and remineralised enamel *in vivo* study. (**a**) Acid-etched enamel in blank control group. (**b**) The magnified micrograph of (a). (**c**) Remineralised enamel after 3 h remineralisation in group A. (**d**) The magnified micrograph of (c). (**e**) emineralised enamel after 5 h remineralisation in group A. (**f**) The magnified micrograph of (e). (**g**) Remineralised enamel after 3 h remineralisation in group B. (**h**) The magnified micrograph of (g). (**i**) Remineralised enamel after 5 h remineralisation in group B. (**j**) The magnified micrograph of (i).

*In vivo* study, the SEM results was well corresponded with that *in vitro*. After treating with 37% phosphoric acid gel, the surface of acid-etched enamel was uneven, and needle-like crystals exposed (Fig. 2a,b). As the remineralisation duration increased, more and more new crystals formed. The acid-etched enamel was completely remineralised at the end of 5 h remineralisation, both in group A with the aid of 1.0 mA and in group B with the aid of 0.5 mA. And their acid-etched profiles were totally invisible (Fig. 2e,f,i,j).

**Figure 3:**
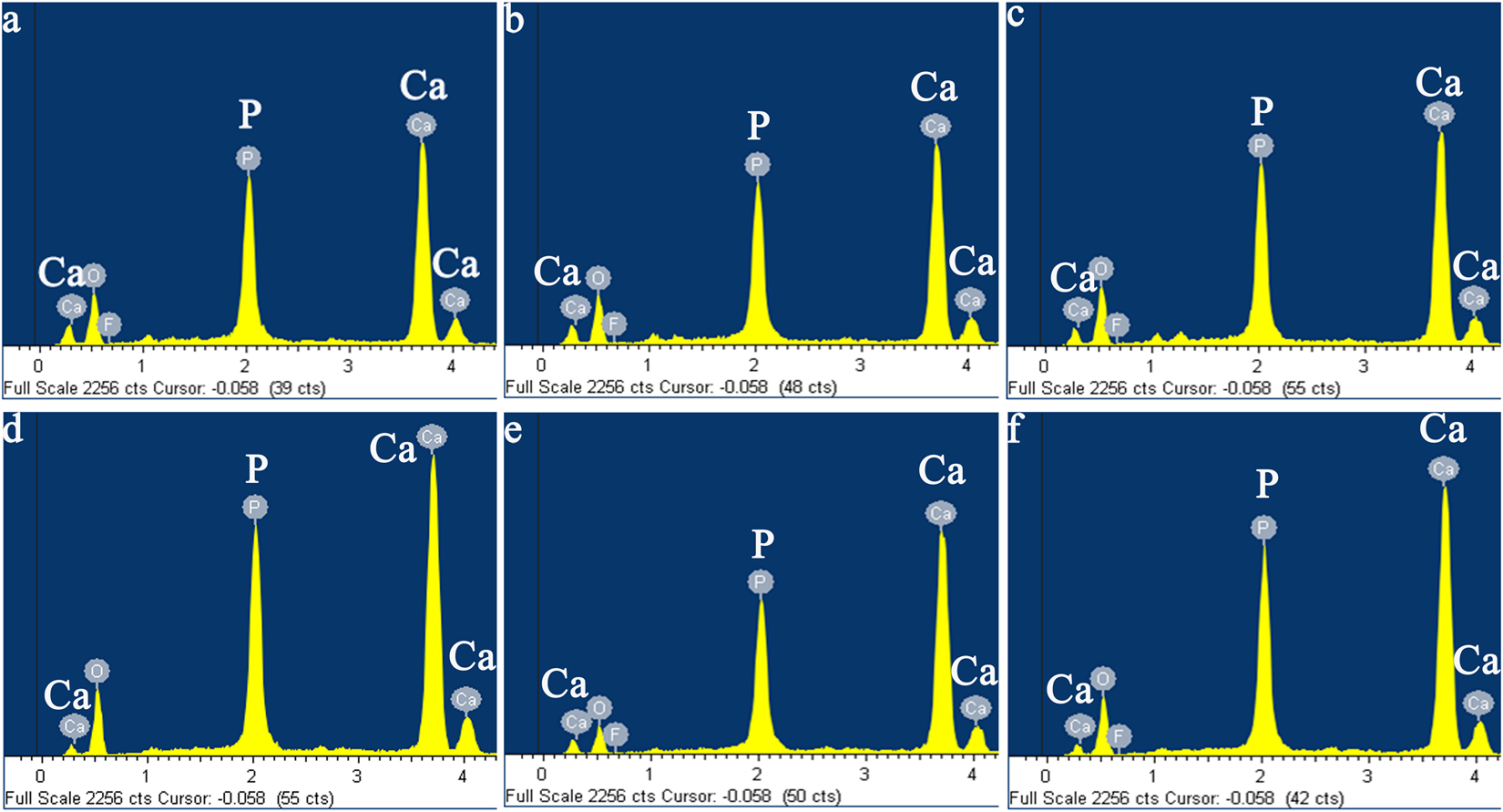
EDS spectra of the remineralised enamel (**a**,**b**) and (**c**) EDS spectra of the remineralised enamel after 3 h remineralisation in experimental group, group A and group B, respectively (**d**,**e**) and (**f**) EDS spectra of the remineralised enamel after 5 h remineralisation in experimental group, group A and group B, respectively.

Figure 3 showed the EDS curves of remineralised enamel *in vitro* and *in vivo* study. These remineralised enamel surfaces had similar chemical compositions with major components of calcium (Ca), phosphorus (P), and oxygen (O). Meanwhile, the weight percentage of calcium was increased with remineralisation duration and current strength, suggesting an increase of mineral contents (Table 1). With the aid of 1.0 mA electric current, after 5 h remineralisation, the Ca/P ratio of remineralised enamel was 1.60 and 1.72 in experimental group *in vitro* study, and in group A *in vivo* study, respectively. Those were consistent with the Ca/P ratio in native enamel. This implied that the newly formed crystals on acid-etched enamel were hydroxyapatites.

**Table 1:** The calcium (weight %), phosphate (weight %) and Ca/P ratio in remineralised enamel.

Duration of Remineralisation	Experimental Group			Group A			Group B		
	Ca	P	Ca/P	Ca	P	Ca/P	Ca	P	Ca/P
3 h	36.89	20.06	1.42	37.57	19.79	1.47	35.70	19.89	1.37
5 h	39.00	20.15	1.60	46.98	21.10	1.72	39.30	19.68	1.54

### The assessment of micro-hardness of remineralised enamel

The Knoop hardness values of native, acid-etched and remineralised surface of enamel were shown in Table 2. *In vivo* study after 5 h remineralisation, both the values of the micro-hardness of remineralised enamel in group A with the aid of 1.0 mA (p < 0.001, two sample T test) and in group B with the aid of 0.5 mA (p < 0.001, two sample T test) were significantly different from that of acid-etched enamel. There was no significant difference in micro-hardness among remineralised enamel in group A with aid of 1.0 mA and in group B with aid of 0.5 mA, and native enamel (p = 0.057, One way ANOVA).

**Table 2:** The micro-hardness value of native, acid-etched and remineralised enamel.

Sample	Mean Knoop hardness value (KHN) ± standard deviation (S.D.)
Natural Enamel	343.73 ± 21.45
Acid-etched enamel	66.55 ± 19.25
Remineralised enamel in group A for 5 h remineralisation	322.55 ± 31.90
Remineralised enamel in group B for 5 h remineralisation	305.68 ± 40.05

### The evaluation of rabbit dental pulp vitality

**Figure 4:**
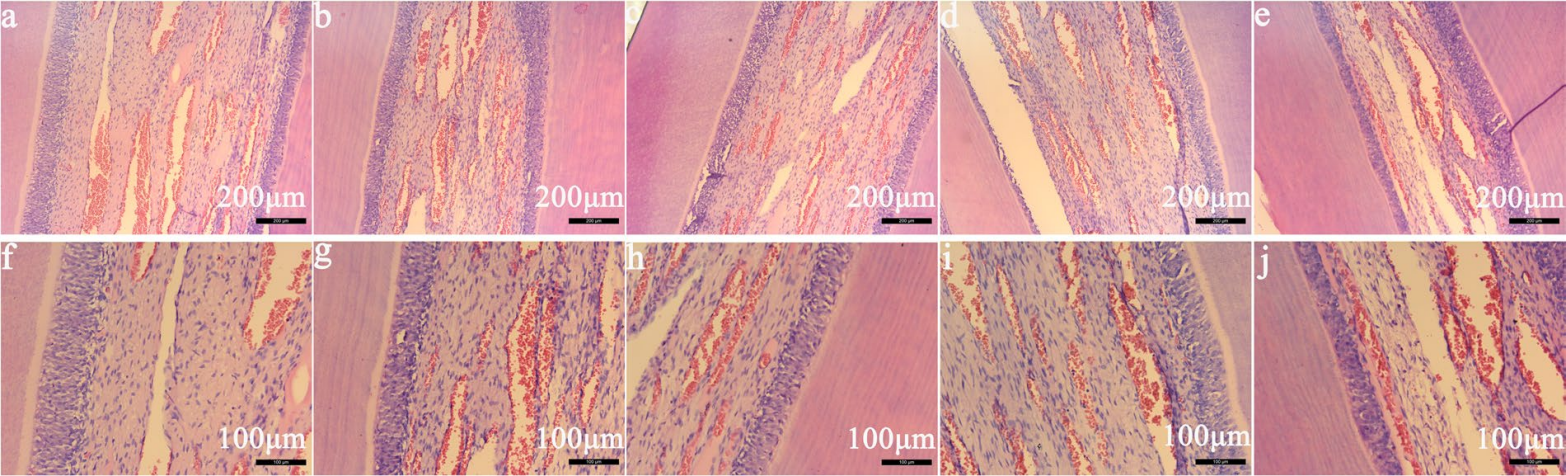
Histological section photomicrographs *in vivo* study. (**a**,**b**) Histological photomicrograph after 3 h remineralisation, in group A and group B respectively. (**c**,**d**) Histological photomicrograph after 5 h remineralisation, in group A and group B respectively. (**e**) Histological photomicrograph in blank control group. (**f**–**j**). The magnified photomicrograph of (**a**–**e**), respectively.

Figure 4 illustrated the histological evaluation of rabbit healthy dental pulp, and rabbit dental pulp in group A with the aid of 1.0 mA and in group B with the aid of 0.5 mA *in vivo* study, after 3 h and 5 h remineralisation. It was found that, after 3 h and 5 h remineralisation both in group A with the aid of 1.0 mA and in group B with the aid of 0.5 mA, the wall of vessels of rabbit dental pulpal tissue was intact, and all odontoblasts appeared in the palisade pattern that was similar to the observations in rabbit healthy dental pulp. There was no evidence of disturbances of odontoblasts, vascular collapse, or the serious acute inflammatory reaction detected in rabbit dental pulp *in vivo* study.

## Discussion

A novel remineralisation system in managing enamel demineralisation was offered by incorporating CPP-ACP with electrophoresis in this paper. Both *in vitro* and *in vivo* study, it was successfully proved that electrophoresis could effectively accelerate the remineralisation speed of CPP-ACP and strengthen its repair effects on demineralised enamel. In the electrophoresis-aided system, due to the hydrolysis reaction in cathode, it could result in the increase of local pH value. Since CPP is negatively charged around neutral pH^23^, the electrochemical reaction in cathode could make CPP-ACP nanocomplexes being negatively charged. Thereafter, the electrokinetic motion of negatively charged CPP-ACP nanocomplexes was initiated by the influence of direct electric field, which was to accelerate the immigration of negatively charged CPP-ACP nanocomplexes from the cathode to demineralised enamel surface. This would be beneficial to the increase the remineralisation speed of CPP-ACP. Additionally, the increasing pH value would attract more calcium phosphate binding to CPP, and provide more bioavailable calcium phosphate for demineralised enamel lesions^24^.

In the study of Wu, it was identified that electrophoresis could effectively accelerate the mineralisation speed^25^. The linear velocity in the mineralisation areas caused by electrophoresis was shown to be approximately 15 times greater than that caused by simple diffusion^26^. In this study, it was observed that, with the aid of electrophoresis, it could effectively accelerate the mineralisation speed of CPP-ACP and promote the remineralisation of demienralised enamel. After 5 h remineralisation in control group, the amount of demineralised crystals was exposed and the profile of demineralised enamel still could be detected. With the aid of electrophoresis, the demineralised enamel was fully and completely remineralised at the end of 5 h, based on the SEM observations *in vitro* (Fig. 1g) and *in vivo* studies (Fig. 2e,i). The EDS results demonstrated that the weight percentage of calcium in remineralised enamel increased with the remineralisation duration (Table 1). Theoretically, in the same remineralisation duration, the higher electric current applied in group A with the aid of 1.0 mA should have led to faster electrokinetic motion of negatively charged CPP-ACP nanocomplexes and higher mineral content in remineralised enamel than that in group B with the aid of 0.5 mA. However, both in the SEM and EDS results, the remineralisation effects in remineralised enamel between group A and group B was not significantly different. This might be caused by insufficient disparity between 1.0 mA and 0.5 mA to detect the influence of current strength on remineralisation kinetics of CPP-ACP.

Enamel is the outlayer of enamel that is composed of more than 95% mineral content and less than 1% organic components. During the process of native enamel formation, the protein-protein interactions and protein-mineral interaction play a crucial role in regulating the nucleation and organisation of mineral phases^27^. In the matrix protein of enamel, amelogenin takes up about 90%^1,28^. In the demineralised enamel, crystals are charged with electrics, which make them be able to directly attract calcium and phosphate ions from remineralisation solution for reducing surface energy. This can lead to a self-growth of original crystals in enamel^29^, corresponding with the SEM observations in Fig. 1f. The fragmentary crystals in demineralised enamel started to fuse together after 3 h remineralisation, and resulted in a lengthening and thickening growth of original crystals (Fig. 1f, arrow). Like amelogenin in enamel, the phosphophoryn in dentine also can regulate the crystal nucleation and growth. Evidence shows that the aspartic-serine-serine in dentine phosphoprotein is able to promote the formation of crystals and improve the mechanical properties of acid-etched human enamel^30^. However, neither amelogenin- or phosphoprotein-system was applied in the current study. Electrophoresis was also proved to be capable to effectively promote the crystal formation. In the study of Watanabe, electrophoresis was used to precipitate hydroxyapatites in an agarose gel to form a hydroxyapatite/agarose composite. It was found that, with the aid of electrophoresis (100 V), the complete mineral formation was obtained in 30min^26^. In the SEM micrographs of remineralised enamel in experimental group after 3 h remineralisation *in vitro* study, it showed that the boundary between new crystal layer (Area I) and original enamel (Area II) (Fig. 1e). Newly formed crystals were clustered and densely packed on the surface of demineralised enamel at the end of 3 h remineralisation (Fig. 1e, area I). In the area of original enamel, not only the self-growth of demineralised enamel was observed, the formation of new crystals was also detected (Fig. 1f, rectangle). With the increased remineralisation time, more and more new crystals have produced and gathered to form a thick layer of crystals covering on the surface of demineralised enamel. At the end of 5 h remineralisation, demineralised enamel was fully remineralised, and its demineralised profile completely disappeared (Fig. 1g).

CPP can stabilise amorphous calcium phosphate at the tooth surface, and increase the local level of calcium phosphate concentration. Thus, CPP-ACP, as a calcium phosphate reservoir, could maintain a supersaturation status of calcium and phosphate ions in the oral environment, which is beneficial to the induction of remineralisation^31^. Numerous studies have identified that CPP-ACP owns the ability of promoting remineralisation and preventing demineralisation for enamel lesions^7,14,32^. However, the remineralisation potential of CPP-ACP regarding its re-hardening effects on demineralised enamel has been questioned in the literatures^17,33,34^. In the study of Zheng *et al*., it was indicated that the mineral deposited on remineralised enamel was mainly amorphous calcium phosphate (ACP), which was the reason why the mechanical properties of remineralised enamel was weak^17^. Obviously, ACP is different from the hydroxyapatite which is the microstructure in native enamel. The highly oriented hydroxyapatites play an important role in sustaining the excellent mechanical and anti-wear property of enamel, and preventing dentinal tubules away from external stimuli^35^. In this study, the EDS results *in vitro* and *in vivo* study both showed that, after treating with electrophoresis-aided CPP-ACP remineralisation system, the newly formed crystals on demineralised enamel were hydroxyapatites. Moreover, the micro-hardness of acid-etched enamel in group A and B was successfully recovered up to the value of native enamel (p > 0.05). In addition, the new crystal layer formed on demineralised enamel was not affected by 2 min ultrasonic treatment (40 KHz, 407 W), implying the bonding surface between new crystals and demineralised enamel was reliable^36,37^. Bonding surface is very important in dental restorative materials. Poor bonding surface will lead the occurrence of leakage and insufficient adhesion that increases the risk of bacteria invasion and secondary caries, and eventually results in the failure of restoration. In this study, the strong bonding between newly grown layer and demineralised enamel surface could effectively prevent the formation of secondary caries.

To date, no evidence has been available to show the effect of electric field on pulp vitality due to methodological difficulties. Based on the histological results in this study, it demonstrated that both 1.0 mA and 0.5 mA produced no harm on rabbit dental pulp. Thus, the electrophoresis-aided CPP-ACP system used in this study was proved to be safe to apply at dental clinics.

## Methods

### The preparation of CPP-ACP suspension

CPP-ACP suspension was prepared by mixing 1 g CPP-ACP paste (Tooth Mousse, GC Corp., Tokyo, Japan) with 1 mL remineralisation solution (2.58 mM CaCl_2_·2H_2_O, 1.55 mM KH_2_PO_4_, 50 mM trihydroxymethylaminomethane (Tris)-hydrochloric, 180 mM NaCl and 1 mg/L NaF) at pH 7.6. NaCl agarose gel was prepared by mixing 1.0 g agarose powder into 100 mL 0.9% NaCl solution and heated into 100 °C until completely dissolution of agarose powder.

### *In vitro*

#### Specimens preparation

This study was approved by The University of Hong Kong/Hospital Authority Hong Kong West Cluster Institutional Review Board (IRB UW17-009) and was carried out in accordance with approved guideline for research involving human subjects. Extracted third molars with sound enamel were obtained from participants with patients’ written informed consent. And all methods were carried out in accordance with relevant guidelines and regulations. From these extracted teeth, twelve teeth with similar size were chosen, cleaned with normal saline to remove the dribs, and fixed in 10% buffered formalin (Sigma-Aldrich, St. Louis, MO, USA) for at least 3 months.

#### Lesion formation and pH cycling

Except for the lingual surfaces, teeth were varnished with an acid-resistant nail polish, and then placed in a demineralisation solution (2.2 mM CaCl_2_·2H_2_O, 2.2 mM KH_2_PO_4_, 50 mM acetate, pH 4.4) for 96 h at 37 °C to create lesions of 70–100 μm deep. Following lesion development, the fragments were rinsed thoroughly with deionised water. After that, teeth were pH cycled at room temperature through a 30 min immersion in demineralisation solution (1.5 mM CaCl_2_, 0.9 mM KH_2_PO_4_, 50 mM acetate) at pH 5.0 followed by a 10 min immersion in remineralisation solution (20 mM 4-(2 hydroxyethyl)-1 piperazineethanesulfonic acid (HEPES), 1.5 mM CaCl_2_, 0.9 mM KH_2_PO_4_, 150 mM KCl) at pH 7.0. The procedure was performed six cycles per day for 8 days. All of the solutions were freshly prepared for use in the experiment. Teeth were kept in deionised water at 4 °C overnight. At the end of the cycling period, all specimens were washed with deionised water, and air dried^38^.

**Figure 5:**
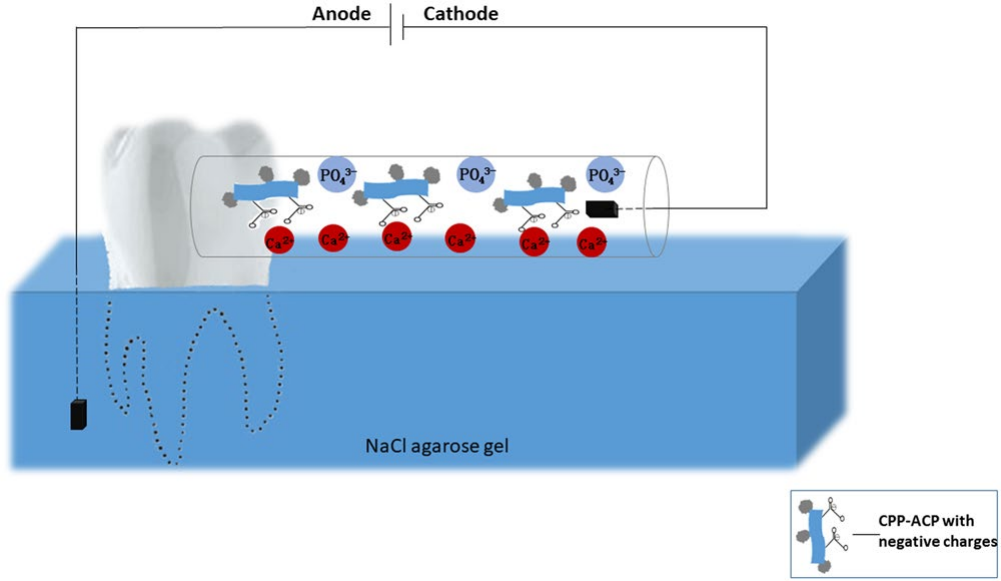
The model of electrophoresis-aided CPP-ACP remineralisation system *in vitro* study.

Twelve demineralised teeth were equally allocated into experimental group (n = 6), and control group (n = 6). In experimental group, teeth were remineralised by the electrophoresis-aided remineralisation device, which consisted of a two-way horizontal polyether tube, a plastic cell and two electrodes. First, root of the tooth was fixed into the plastic cell filled with NaCl agarose gel, and its crown was exposed outside of the cell. Then, the two-way horizontal polyether tube was filled with CPP-ACP suspension. Next, one side of polyether tube was connected with the demineralised enamel surface in tooth, and another side was connected with cathode. Lastly, the anode was set into the bottom of the cell, as shown in Fig. 5. The electric current was maintained constant at 1.0 mA and controlled by electrophoresis device (DYY-10C Electrophoresis, Liuyi Instrument Factory, Beijing, China). The CPP-ACP suspension and NaCl agarose gel were refreshed every hour. In control group, teeth were demineralised by CPP-ACP suspension only. In experimental group and control group, tooth was demineralised for 3 h or 5 h. At the end of each remineralisation process, tooth was cleaned by ultrasonic treatment (40 KHz, 407 W) for 2 min and then stored at 4 °C for further characterisation.

### In vivo

#### Preparation of demineralised enamel surface on rabbit’s incisors

Ten male New Zealand rabbits (age: 90–120 days and body weight: 2.065–2.309 kg, the Department of Animal Experimental Centre of Anhui Medical University (certificate number SYXK (Wan) 2013-004)) were used in this study. All animal experimental procedures were approved by the Anhui Medical University Experimental Animal Ethics Committee and carried out in accordance with the National Institutes of Health guide for the care and use of Laboratory animals (NIH Publications No. 8023, revised 1978). ARRIVE guidelines were followed. The rabbits were initially anesthetised with an intravenous injection of pentobarbital sodium (Sigma-Aldrich, St. Louis, MO, USA) at a dose of 30.0 mg/kg. For maintaining the rabbits in a state of anesthesia, a supplementary does of 10 mg/kg was given as needed during the experiment^39^.

#### The design of a personalised mold for loading CPP-ACP suspension

**Figure 6:**
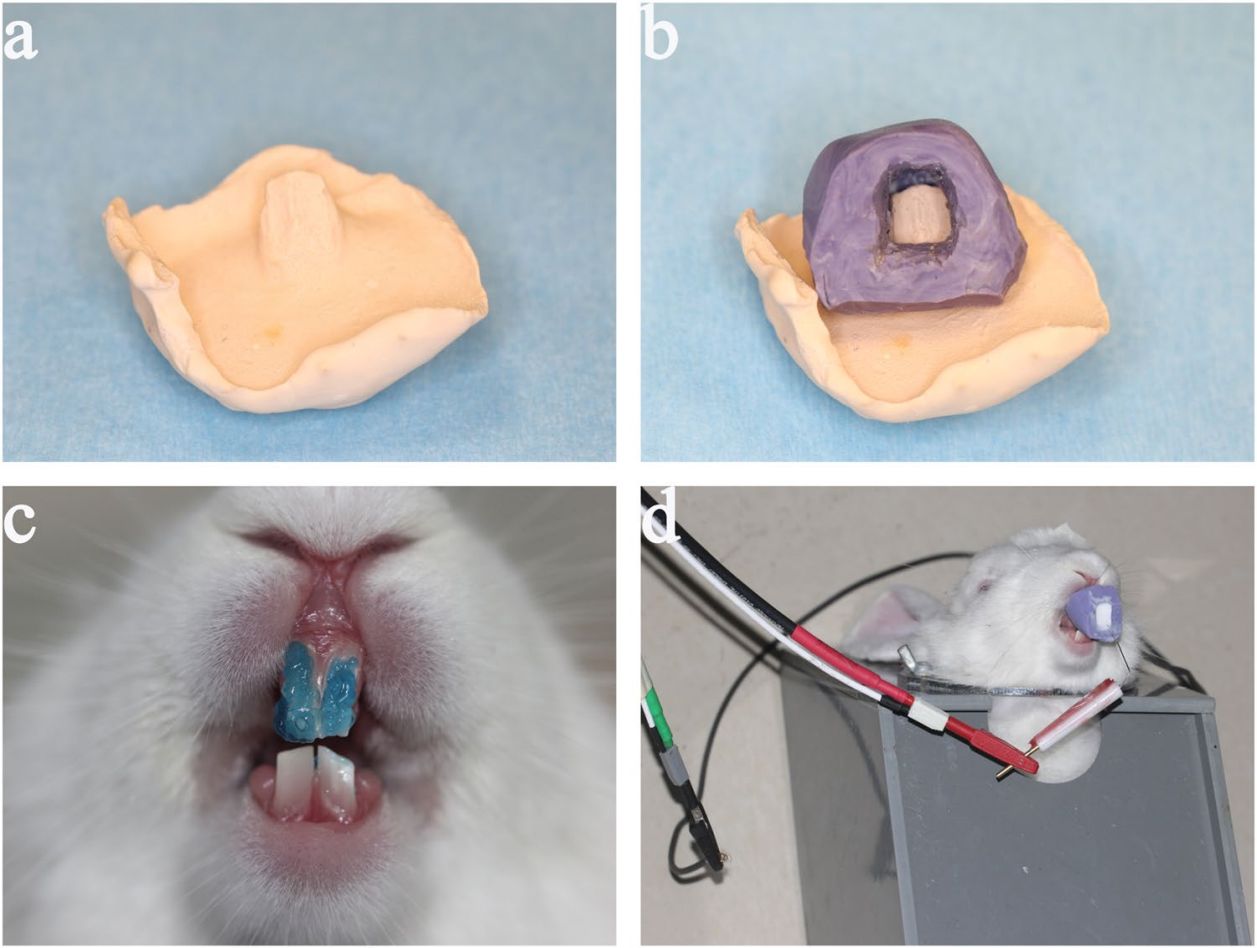
Electrophoresis-aided CPP-ACP remineralisation system on acid-etched rabbit enamel. (**a**) The plaster working model. (**b**) The second impression on the plaster model. (**c**) The frontal view of enamel covered with 37% phosphoric acid. (**d**) The assembling of electrophoresis-aided CPP-ACP remineralisation system.

A personalised mold with an opening cavity on the labial surface was made to load CPP-ACP suspension. First, the alginate impressions were taken from the rabbit maxillary incisors. Then, plaster working model was primed and shaped (Fig. 6a). Next, the second impression (Fig. 6b) was made on the plaster model by using the heavy body of hydrophilic addition-type silicone rubber impression materials (3 M ESPE, Seefeld, Germany). Lastly, the personalised mold with an opening cavity on the labial surface was formed by removing labial silicone rubber of the second impression.

#### The preparation of acid-etched enamel surface on rabbit’s maxillary incisors

The labial surface of the maxillary incisor was etched with 37% phosphoric acid gel (Gluma Etch 35 Gel, Heraeus Kulzer GmbH, Germany) (Fig. 6c) for 15 s and rinsed with a large amount of deionised water to form an acid-etched enamel surface. Ten rabbits with acid-etched enamel were randomly allocated into experimental group remineralising by electrophoresis-aided CPP-ACP (group A treated with 1.0 mA (n = 4); group B treated with 0.5 mA (n = 4)), and blank control group without remineralisation (n = 2).

#### Assembling the remineralising appliance on rabbit acid-etched incisors

The rabbits with acid-etched enamel in group A (treated with 1.0 mA) and group B (treated with 0.5 mA) were treated with a two-electrode system under galvanostatic condition. The anode of the two-electrode system was connected with the skin of rabbit’s head, and its cathode was inserted into the personalised mold loaded with CPP-ACP suspension (Fig. 6d). The electric current applied in group A and B were respectively 1.0 mA and 0.5 mA, controlling by electrochemical workstation (VersaSTAT3, AMETEK Inc., America). In group A (treated with 1.0 mA) and group B (treated with 0.5 mA), the rabbit was remineralised for 3 h or 5 h. At the end of each remineralisation process, the maxillary incisors were extracted. One of two maxillary incisors in each rabbit was cleaned by ultrasonic treatment (40 KHz, 407 W) for 2 min, dehydrated with gradual ethanol and dried in the critical evaporator for further characterisation; the another one was fixed in 10% formalin for histological evaluation.

### The characterisation and evaluation of remineralised enamel

For characterision of crystal morphology and element compositions, new crystals formed on remineralised enamel were evaluated by field-emission scanning electron microscopy (SEM) and energy dispersive spectroscopy (EDS) (Hitachi S4800, Hitachi Ltd., Tokyo, Japan; FEI, Sirion 200, USA), respectively. For evaluation the mechanical properties of remineralised enamel, a micro-hardness tester (Leica DC 300, Leitz, Germany) was used to measure the micro-hardness of remineralised enamel. Before testing, the Knoop tip was calibrated with a standard calibration reference block. Six test points with 30 mm spacing were performed on the surface of the sample. Data were recorded and analysed by statistic software (SPSS 24, IBM). Differences were considered significant at p < 0.05. Data were expressed as mean ± SD.

### The evaluation of the safety of electric field on rabbit dental pulp

All specimens were decalcified in 15% EDTA for three weeks. After decalcification, specimens were dehydrated gradually, cleaned and embedded in paraffin. Every specimen was cut into 5-μm serial sections along axis in the buccolingual direction, mounted on glass slides and subjected to hematoxylin and eosin. All sections were blindly evaluated by two experienced oral and maxillofacial pathologists using the optical microscope (H600L, Nikon, Japan) under ×20 and ×50 magnifications.

## Conclusion

Both *in vitro* and *in vivo* study, electrophoresis could effectively accelerate the remineralisation kinetic of CPP-ACP and strengthen its re-hardening effects on demineralised enamel. Electrophoresis-aided CPP-ACP was a safe and efficient option in managing enamel demineralisation.

### Data availability statement

The datasets generated and analysed during this study are available from the corresponding authors on reasonable request.

## Electronic supplementary material

Supplementary file
