# Supplementary material for: In vitro and in vivo evaluation of electrophoresis-aided casein phosphopeptide-amorphous calcium phosphate remineralisation system on pH-cycling and acid-etching demineralised enamel
Source: Sci Rep. 2018 Jun 11;8:8904. doi: 10.1038/s41598-018-27304-5 (PMC5995962; doi:10.1038/s41598-018-27304-5)
Supplement: Supplementary file 1 — Supplementary file [file 41598_2018_27304_MOESM1_ESM.docx]

**In vitro and in vivo evaluation of electrophoresis-aided casein phosphopeptide-amorphous calcium phosphate remineralisation system on pH-cycling and acid-etching demineralised enamel**

Yu Yuan Zhang ^1^, Hai Ming Wong ^1*^, Colman PJ McGrath ^1^, Quan Li Li ^2^

^1^Faculty of Dentistry, The University of Hong Kong, 34 Hospital Road, The Prince Philip Dental Hospital, Hong Kong;

^2^Department of Prosthodontic, Collage and Hospital of stomatology, Anhui Medical University, China.

**Corresponding authors**

Hai Ming Wong, Faculty of Dentistry, The University of Hong Kong, 34 Hospital Road, The Prince Philip Dental Hospital, Hong Kong; Email: [wonghmg@hku.hk](mailto:wonghmg@hku.hk); Tel No: 852 28590261; Fax No: 852 25593803

Quan Li Li, Department of Prosthodontic, Collage and Hospital of stomatology, Anhui Medical University, No. 69, Meishan Road, Heifei, China. Email: [ql-li@126.com](mailto:ql-li@126.com); Tel No: +86 0551 5118677; Fax No: +86 0551 5111538

**Supplementary text**

**The characterisation and evaluation of remineralised enamel after 8h remineralisation in vitro and in vivo study**

According to the SEM micrographs, after 8h remineralisation the profile of demineralised enamel in vitro and in vivo study could not be detected. The surfaces were fully covered with a thick layer of crystals (Supplementary Fig.1). The EDS curves of remineralised enamel had similar chemical compositions with major components of calcium (Ca), phosphorus (P), and oxygen (O) (Supplementary Fig.2). The Ca/P ratio of remineralised enamel in experimental group in vitro study, in group A and group B in vivo study, was 1.60, 1.64 and 1.69, respectively (Supplementary Table 1). These were corresponding with the Ca/P ratio in native enamel (1.67). This implied that the newly formed crystals on acid-etched enamel were hydroxyapatites. The micro-hardness of remineralised enamel in group A (370.43 + 26.70) and group B (337.54 + 29.23) after 8h remineralisation in vivo study was not significantly different from that in native enamel (p=0.283, One-way ANOVA; Supplementary Table 2). That means after 8h remineralisation, the micro-hardness of acid-etched enamel in experimental group, group A and group B all recovered up to the level of native enamel (p>0.05).

**Supplementary Figures and Tables**


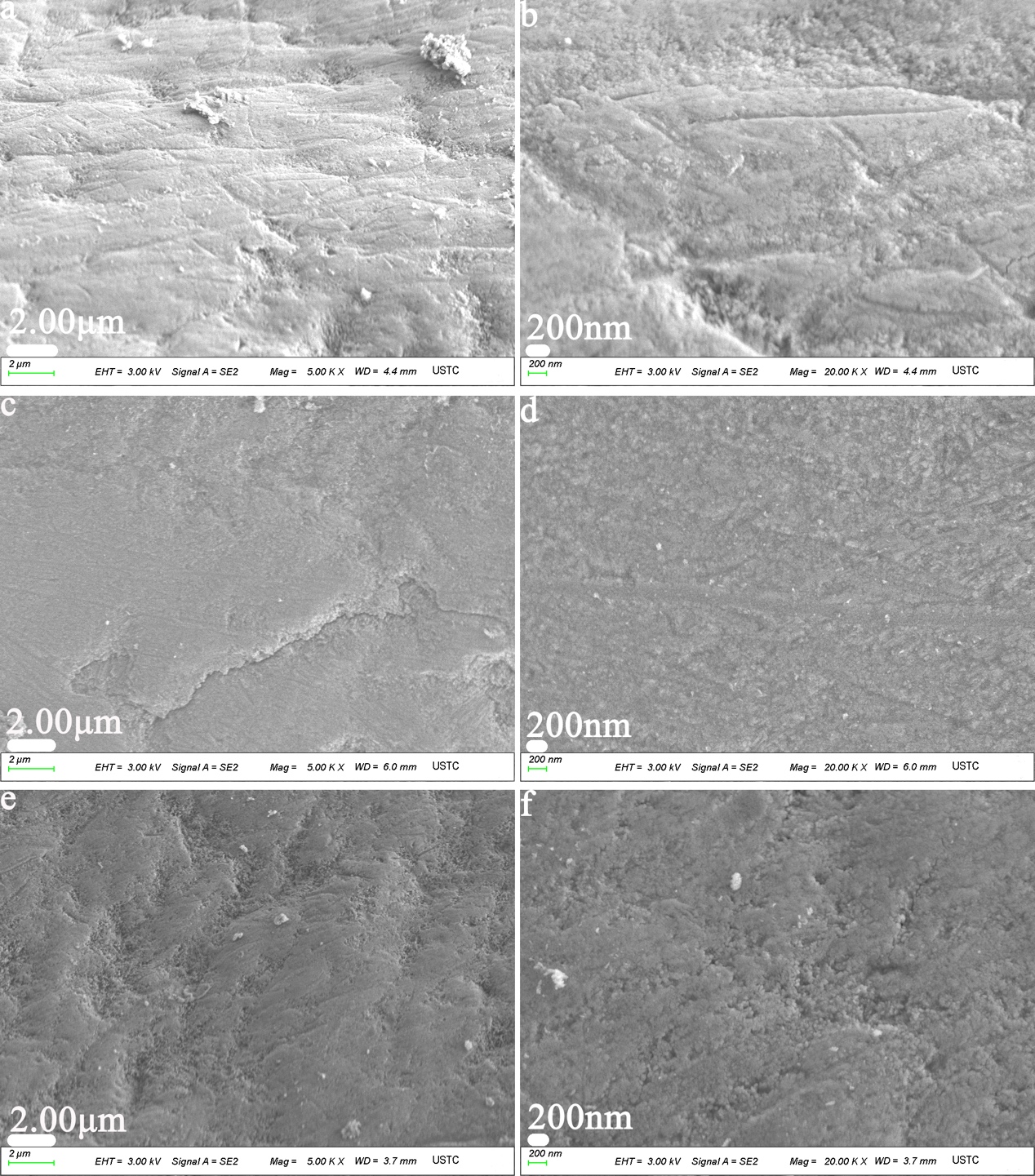


**Supplementary Fig. 1 SEM micrographs of remineralised enamel after 8h remineralisation**

(a) Remineralised enamel in experimental group in vitro study;

(b) The magnified micrograph of (a);

(c) Remineralsied enamel in group A in vivo study;

(d) The magnified micrograph of (c);

(e) Remineralsied enamel in group B in vivo study;

(f) The magnified micrograph of (e).


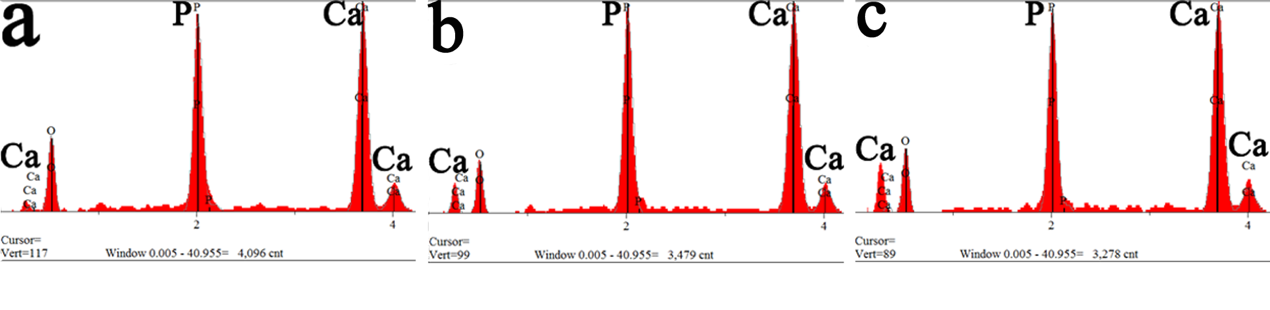


**Supplementary Fig. 2 EDS spectra of the remineralised enamel after 8h remineralisation**

(a), (b) and (c) EDS spectra of the remineralised enamel in experimental group, group A and group B, respectively;

**Supplementary Table 1**

**The calcium (weight %), phosphate (weight %) and Ca/P ratio in remineralised enamel**

| **Duration of Remineralisation** | **Experimental Group** | | |  | **Group A** | | |  | **Group B** | | |
| --- | --- | --- | --- | --- | --- | --- | --- | --- | --- | --- | --- |
|  | **Ca** | **P** | **Ca/P** |  | **Ca** | **P** | **Ca/P** |  | **Ca** | **P** | **Ca/P** |
| **8h** | 50.93 | 24.78 | 1.60 |  | 55.83 | 26.51 | 1.64 |  | 54.47 | 24.86 | 1.69 |

**Supplementary Table 2**

**The micro-hardness value of native, acid-etched and remineralised enamel.**

| **Sample** | **Mean Knoop hardness value (KHN)**  **+ standard deviation (S.D.)** |
| --- | --- |
| Natural Enamel | 343.73 + 21.45 |
| Acid-etched enamel | 66.55 + 19.25 |
| Remineralised enamel in  group A for 8h remineralisation | 370.43 + 26.70 |
| Remineralised enamel in  group B for 8h remineralisation | 337.54 + 29.23 |
